# Aerobics, Quality of Life, and Physiological Indicators of Inactive Male Students’ Cardiovascular Endurances, in Kashan

**Published:** 2014-06-15

**Authors:** Mohammad Ebrahim Bahram, Gudarz Akkasheh, Negar Akkasheh

**Affiliations:** 1 Department of Exercise Physiology, Faculty of Physical Education and Sports Sciences, Isfahan University, Isfahan, IR Iran; 2 Department of Psychiatry, Kashan University of Medical Science, Kashan, IR Iran; 3 Faculty of Medicine, Semnan University of Medical Sciences, Kashan, IR Iran

**Keywords:** Quality of Life, Cardiovascular Endurance, Aerobic Exercise, Inactive

## Abstract

**Background:**

Studies show that lack of exercise and physical activity during childhood and teenage years is directly related to different diseases in adulthood.

**Objectives:**

The main purpose of this study was to investigate the effects of an eight-week aerobic exercise on the quality of life as well as physiological indicators of cardiovascular endurance of inactive high school male students in Kashan.

**Materials and Methods:**

The study was a field trial using pretest and post-test. Three hundred high school male students in Kashan, Iran, were recruited and interviewed by the researchers, using a questionnaire. Of the inactive ones, 30 who reached the highest criteria standards, were selected as samples and randomly divided to two equal groups. The maximum consumed oxygen (VO_2_max) and resting heart rate were measured by Quinn aerobic test, and the quality of life was measured by the World Health Organization Quality of Life (WHOQOL-26-Breef) questionnaire. The exercise program included an eight-week aerobic exercise, three times per week, with 60%-75% of the maximum heart beat. During the exercise, the subjects had no other sport activity. To check the normal distribution of the data, Kolmogorov-Smirnov test was used. To evaluate the pretest and post-test results, paired t-test was used and for comparing the groups, independent t-test was applied. All the analyses were performed by SPSS software version 16.

**Results:**

The mean ages of intervention and control groups were 17.46 ± 1.30 and 17.53 ± 1.18, respectively. The mean weight of the intervention group was 56.73 ± 9.91 kg and its mean body mass index (BMI) was 19.88 ± 3.42. In the control group, the mean weigh and BMI were 60.06 ± 11.96 kg and 20.79 ± 3.51, respectively. The quality of life and its components improved significantly in the intervention group (physical (P = 0.0001), mental (P = 0.0001), social (P = 0.0001), and environmental (P = 0.0001) aspects). VO_2_max (P = 0.001) and the resting heart beat (P = 0.0001) significantly improved in the intervention group. No significant difference was observed in the control group (P ≥ 0.05).

**Conclusions:**

Aerobic exercise program improved the quality of life as well as the physiological indicators. Physical activities can be used as both appropriate model and nonpharmaceutical approach to prevent and cure some diseases.

## 1. Background

Today, paying attention to quality of life as well as health promotion and disease prevention is considered as a national priority. Not only the scientific, specialized, and clinical communities, but also the public, has fully been aware of the vast influence of lifestyle on physical and mental health (1-5). A life style accompanying with exercise has a direct effect on reducing cardiovascular diseases, cancer, depression, anxiety, articular diseases and diabetes (6-9). In the last 50 years, epidemiological studies have shown that lack of movement and low physical preparation in children and teenagers have been related to deaths, cardiovascular diseases, and diabetes in adulthood (10, 11), whereas those physically active are less exposed to diseases and survive longer. Studies in this regard show that gaining weight, obesity, and physical deformities due to lack of physical activities are increasing in Iranian students, leading to many problems. In addition, there is a relation between cardiovascular risk factors with cardiorespiratory preparation that reduces the quality of life (12, 13). A study showed that 20 minutes of aerobic exercise (running) with maximum heart beat of 60 to 80 for six weeks, three to five times a week, can increase aerobic endurance, reduce fat, and improve social interactions (14). A study on the effects of aerobic exercise on high school inactive students suffering from being overweight, also showed decrease of fat and increase of consumed oxygen (15, 16). Another research investigated the effects of exercise on quality of life during a period of eight weeks, three times a week. The results showed that a fixed regular physical activity can increase the quality of life as well as cardiovascular endurance of the adults from different perspectives (17). Result of the studies showed that children participating in low-frequency exercises had weaker life qualities in comparison with their active peers (17-19). Although, the results of a study illustrated no significant difference in the life qualities of active and inactive children after exercise, while there was a significant difference in their communication skills (20). According to various studies (21-23), different life quality dimensions, weight loss, and social relations, could also be affected after sport exercises. Several other studies also expressed that sport had positive effects on physical, environmental, psychological and social health, as well as leisure time of the students (24-27). Modern life has reduced the exercise and the consequences are obesity, hyperlipidemia and its related diseases.

## 2. Objectives

The aim of this study was to investigate the effects of an eight-week aerobic exercise on the quality of life as well as physiological indexes of cardiovascular endurance of high school inactive students.

## 3. Materials and Methods

### 3.1. Actions

This pilot experimental study using pretest and post-test was conducted in several stages on experimental and control groups through a random systematic procedure. Eight hundred questionnaires were distributed among eight male high schools in Kashan, during the educational years of 2012-2013. The questionnaires contained data such as personal information, disease history, sport background, sport activities, etc. Of the total 800, 620 were collected and 200 subjects were interviewed based on the minimum defined criteria to enter the study. Afterwards, based on the formula, the total number of subjects was divided to sample ones; 30 inactive subjects were selected and divided randomly to two groups. Written informed consents were obtained from all subjects. They were completely healthy and used no medicines. The inclusion criteria were: having no regular sport activities in the last five months; no history of cardiovascular diseases, high blood pressure, high blood cholesterol, smoking, diabetes, heart or brain stroke in the first relatives; no history of disease or taking special drugs. The exclusion criteria contained: infectious diseases or musculoskeletal problems during the study; using ergogenic drugs; parents' disagreement.

### 3.2. Measurement Tools and the Procedure

For assessment and selection of samples, the researcher made a questionnaire including demographic and personal characteristics, past medical history, family history, exercise and its levels, and medication. This questionnaire was verified by experts. The subjects’ heights and weights were measured using a digital scale tape (BEURER PS06M42, Germany) and measurements of height and weight is done with lowest clothing level such as shorts. body mass index (BMI) was calculated through this formula:

Weight/squared height (kg/m^2^)

Cardiovascular endurance indices were measured using Quinn steps exercise. The 26-item World Health Organization Quality of Life Questionnaire (WHOQOL-26) was used. To measure the cardiovascular endurance via the estimated maximum heart rate and maximum oxygen consumption (VO_2_max), a stepping-like bench, approximately 25/16 inch height (275/41 cm), was used. Each student stepped up and down for about three minutes and finally after five seconds, their heart beats were measured for about 15 seconds from the carotid region. Afterwards, VO_2_max (mL/kg of body weight per minute) was measured through McArdle formula for men (Vo_2_max = 111.13 - (0.42 × HR). In addition, the maximum heart beat was assessed through 220-age formula. The subjects’ life qualities were assessed through 26-item WHOQOL twice before and after the exercises in both control and experimental groups. At the end, the data was extracted. Subjects in the control group had no regular sport activities during the exercise.

### 3.3. Test Reliability

To determine the Quinn's step test reliability, the test-retest method was used and the reliability was 0.98 and 0.91, respectively. WHOQOL-26, validated by Nejat and Montazeri et al. was also used. The inter-cluster correlation and Cronbach's alpha were more than 0.70 in all parts. This questionnaire has high reliability and validity. It is a self-assessment tool which can be completed by the subjects in four physical, psychological, social, and environmental aspects via 24 questions (each aspect contains seven, six, three, and eight questions, respectively). The first two questions have no relation with any of the aspects and generally evaluate health and quality of life. Therefore, this questionnaire contains 26 items, each valued 5-point Likert scale. Higher point in each aspect represents more positive attitude toward life (28).

### 3.4. Exercise Protocol

The practical protocol, contained eight weeks of aerobic exercise through running, three sessions a week with maximum heart beat of 60%-75%. Severity of the sport activities was 60%-65% of the maximum heart beat, increasing progressively. The last session reached 75% of the maximum heart beat. Sport activities were performed during 3-4 pm, ranging from about 30 minutes in the early sessions to 50 minutes in the last sessions. The principle of increasing the overload (including the severity and time of activity), stretching and movement activities to warm up and cool down, and active relaxation between the exercises, were also considered. To reach this, a group of experts evaluated and confirmed the exercise protocol. All exercises were performed in Ketabchi sport center. All exercises were performed under the supervision of a trainer and an exercise physiologist.

#### 3.5. Ethical Considerations

The study was performed according to the Helsinki declaration protocol. Objectives of the study were explained to the students, and informed consents were obtained from all the participants. Students could leave the study at any time. The study was approved by the Vice-Chancellor For Research Ethics Committee of Kashan University of Medical Sciences.

#### 3.6. Statistical Analyses

A descriptive study was performed to analyze the data. The Kolmogorov-Smirnov test was used to identify normal distribution of the data. T-test and paired t-test were used to compare the outcome variables in the groups. In all the analyses, P ≤ 0.05 was considered statistically significant. SPSS package version 16 was used for the analyses.

## 4. Results

The mean ages of intervention and control groups were 17.46 ± 1.30 and 17.53 ± 1.18, respectively. The mean weight of the intervention group was 56.73 ± 9.91 kg and its mean BMI was 19.88 ± 3.42. In the control group, weigh and BMI were 60.06 ± 11.96 kg and 20.79 ± 3.51, respectively. There was no significant difference in weight, height, and age of both control and experimental groups at the beginning of the study (P ≥ 0.05) (Table 1). The results showed that an eight-week aerobic exercise led to a significant difference in the quality of life and physiological cardiovascular endurance of the subjects, in post-test of the intervention group. In other words, an eight-week aerobic exercise increased the VO_2_max to 1.09 ± 0.11 mL.kg/min (P = 0.0001) and reduced the resting heartbeat -2 ± 0.06 beat/min (P ≤ 0.05) (Table 2). Significant differences were observed in various aspects of life quality, such as physical (3.87 ± 0.42), mental (5.14 ± 0.55), social (2.13 ± 0.7), and environmental (5.80 ± 0.45). No significant difference was observed in the control group for all variables in post-test (P ≥ 0.05) (Table 3) (s 1-3).

**Table 1.** Demographic Features of the Intervention and Control Groups ^a,b^

Group	Control, n = 15	Experimental, n = 15	P Value
**Age, y**	17.53 ± 1.18	17.46 ± 1.30	0.885
**Weight, kg**	60.06 ± 11.96	56.73 ± 9.91	0.413
**Height, cm**	169.66 ± 7.84	163.73 ± 4.93	0.699
**BMI, kg/m** ^**2**^	20.79 ± 3.51	19.88 ± 3.42	0.478

^a^ Abbreviation: BMI, body mass index.

^b^ Data are presented as mean ± SD.

**Table 2.** Mean and Standard Deviation of Maximum Oxygen Consumption and Resting Heartbeat in the Intervention and Control Groups, Before and After the Test ^a,b^

Cardiovascular Endurance	Groups	Pretest	Post-test	P Value
**Vo**_**2**_**max mL.kg/min**	experimental	43.86 ± 3.16	46.95 ± 3.27	0.001
	control	43.78 ± 4.18	43.89 ± 3.99	0.8854
**Resting heart beat**	experimental	160.40 ± 7.23	158.40 ± 7.17	0.0001
	control	161.21 ± 9.69	161.20 ± 9.88	0.9953

^a^ Abbreviations: Vo_2_max, maximum oxygen consumption

^b^ Data are presented as mean ± SD.

**Table 3.** Mean, Standard Deviation, and t-test, Four Small Indicators of the Quality of Life in Both Intervention and Control Groups, Pretest and Post-test ^a^

Subscales	Groups	Pretest	Post-test	P Value
**Physical health**	experimental	21.86 ± 3.81	25.73 ± 4.23	0.0001
	control	21.00 ± 2.23	21.46 ± 2.47	0.3386
**Mental health**	experimental	19.86 ± 2.94	24.00 ± 2.39	0.0001
	control	20.73 ± 3.08	21.26 ± 3.32	0.4297
**Social relations**	experimental	11.53 ± 1.18	13.66 ± 1.11	0.0001
	control	11.26 ± 1.48	11.60 ± 1.18	0.1131
**Environmental health**	experimental	29.13 ± 5. 35	33.33 ± 50.80	0.0001
	control	26.26 ± 2.40	26.60 ± 1.78	0.3760

^a^ Data are presented as mean ± SD.

**Figure 1.**
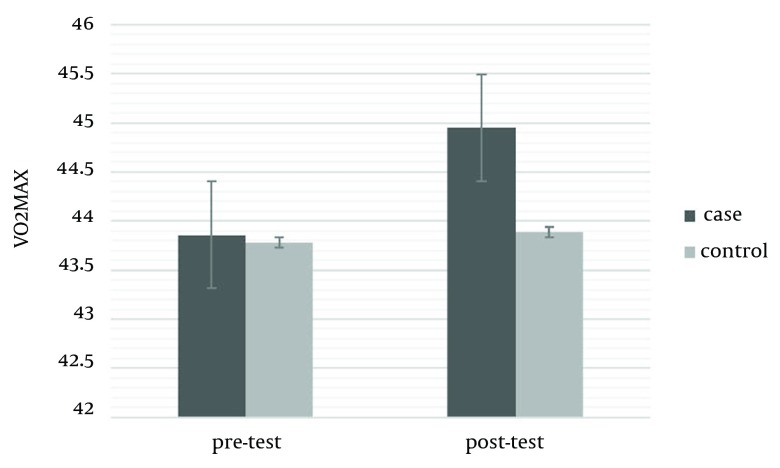
O2 Maximum Volum

**Figure 2.**
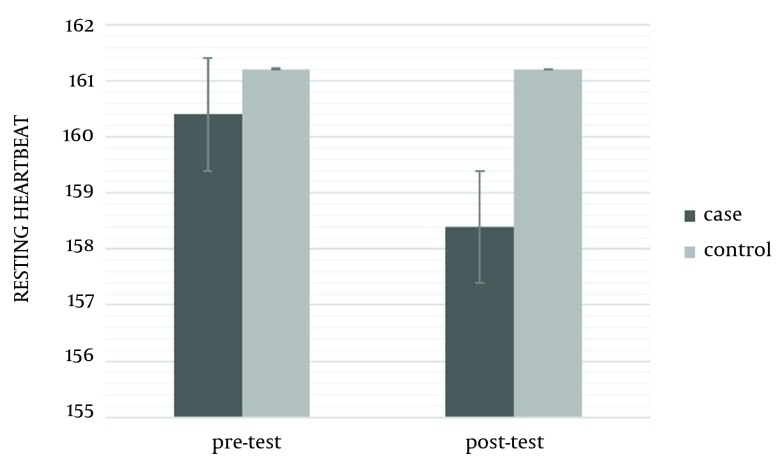
. Heart Beat

**Figure 3.**
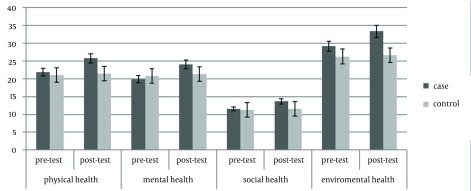
Health Condition

## 5. Discussion

The results of the current study showed significant difference between Vo_2_max and resting heart beat as important indicators of cardiovascular endurance in the intervention group compared with the control group, after eight weeks of aerobic exercise. In addition, the results expressed that aerobic exercises increased physical, mental, social, and environmental indicators of health in inactive young subjects. Some other studies also showed that aerobic exercises could increase the consumed oxygen and reduce heart beat in young inactive subjects and adults, supporting the current findings (15, 17, 18, 19). It seems that increase of Vo_2_max as well as decrease of resting heart beat owing to aerobic exercise, can be due increase in muscle oxidative capacity and total amount of hemoglobin, difference in artery and venous oxygen, increase of end-diastolic volume (cardiac preload), and biochemical processes (18, 19, 29). Aerobic exercises increase the heart potency to pump blood and increase the oxygen consumption in muscles (30). This study showed that selected exercises for about eight weeks with 75% heart beat could provide the necessary motivation for hormone and physiological changes and also increase the fitness indicators. The results of this study were in line with some other similar ones (16, 23-26, 31, 32). secretion of chemical transmitting hormones like serotonin and dopamine, produced as a result of aerobic exercises, are effective in cheerfulness, happiness, self-confidence, and social skills. In addition, sport exercises increase the endorphin level and decrease the cortisol level (a hormone secreted as a result of nervous pressure), which play importants role in improving the mood as well as physical and social health. The results of different studies support our findings (17, 31, 32). On the other side, there were some studies that were not in agreement with ours and showed that sport activities do not affect the quality of life from different perspectives. The reasons for such difference can be found in the type of exercise protocol, severity, and duration of exercises, as well as subjects’ personal characteristics, diet, and sample size (22, 23, 25, 26, 33). The results verified the positive effect of aerobic exercises on the quality of life as well as fitness indicators in inactive students. Regarding the increasing number of inactive students, a general review of sport and educational programs was necessary. Physical activity can be a useful, cheap, and nonoffensive approach for doctors, trainers, behavior experts, and other occupations dealing with human relations, to stay healthy, lead the life with a desirable quality, and prevent and cure psychic disorders. The results necessitated and emphasized on more attention to students' participations in sport activities and physical preparations regarding their health. Hence, it is suggested that authorities conduct similar studies on the effects of different sport activities on different indicators in inactive and overweight boys and girls. They should present proper procedures and increase the quality of training lessons at schools for a healthier society.

However, since the study period was about two months, researchers failed to control the subjects’ nutrition and no considerable weight loss was found in this study.
